# Control Strategy for *Echinococcus multilocularis*

**DOI:** 10.3201/eid1410.080522

**Published:** 2008-10

**Authors:** Daniel Hegglin, Peter Deplazes

**Affiliations:** University of Zurich, Zurich, Switzerland

**Keywords:** Control, Echinococcus multilocularis, praziquantel, urbanization, vulpes, dispatch

## Abstract

*Echinococcus multilocularis*, the causative agent of zoonotic alveolar echinococcosis, can be controlled effectively by the experimental delivery of anthelminthic baits for urban foxes. Monthly baiting over a 45-month period was effective for long-lasting control. Trimonthly baiting intervals were far less effective and did not prevent parasite recovery.

Human alveolar echinococcosis (AE) is a potentially fatal zoonosis (*1*) caused by *Echinococcus multilocularis*, a helminth that is widespread in red foxes (*Vulpes vulpes*). In recent years, fox populations in central Europe have increased and extended their habitats into urban areas (*2*). Consequently, AE rates have increased (*1*). Epidemiologic and ecologic studies have demonstrated that urban peripheries provide suitable conditions for high densities of susceptible final hosts (red foxes) and intermediate hosts (voles) (*3*). Consequently, these densely populated areas can be highly contaminated with *E. multilocularis* eggs and are of special interest for the development of cost-effective control strategies (*2*).

Parasite control by substantially reducing intermediate host density (rodents) or by large-scale fox culling are far less promising strategies than delivering anthelminthic baits for foxes (*4*). In 3 studies from southern Germany, the regular distribution of 15 to 50 praziquantel baits per km^2^ resulted in a significant decrease in the prevalence of foxes from 35%, 64%, and 14–37% to 1%, <20%, and 2%–12%, respectively (*5*–*7*). Different baiting studies in Japan have demonstrated lower environmental contamination with *E. multilocularis* eggs when praziquantel baits are placed around fox dens (*8*), along roads, and in forests that provide protection from the wind (*9*). Even in small-scaled areas of <6 km^2^, anthelmintic baiting has been effective (*10*,*11*), resulting in a lower reinfection rate in foxes because of a lower prevalence of the parasite in intermediate hosts (*11*). However, local eradication of the parasite is difficult to achieve. Long-lasting interventions seem to be necessary for effective control of *E. multilocularis* (*12*). Therefore, cost-effective baiting strategies must be designed.

Our aim was to provide data so that optimal and cost-effective baiting strategies could be designed. To obtain these data, we investigated the effect of different baiting intervals and the postcontrol recovery of the parasite population.

## The Study

**Figure 1:**
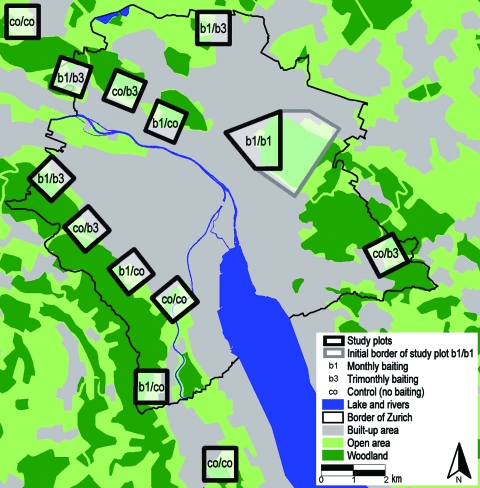
Study area of the anthelminthic baiting experiments in the conurbation of Zurich, Switzerland. Thirteen study plots were defined along the urban periphery during the 2-phased baiting period (phase 1, April 2000–October 2001; phase 2, November 2001–December 2003). Five different treatment schemes were used in these plots: co/co = no bait delivery during the whole study (n = 3 sites of 1 km^2^ ); b1/b3 = bait delivered monthly during the first phase and trimonthly during the second phase (n = 3); co/b3, no bait delivery during the first and trimonthly delivery during the second phase (n = 3); b1/co, monthly bait delivery during the first and no delivery during the second phase (n = 3); b1/b1, monthly bait delivery during the first and the second phase in a single study plot. This largest study plot comprised initially an area of 6 km^2^ (gray line) and finally an area of 2 km^2^ during the second baiting phase.

The study, conducted during 1999 and 2007, was designed as a follow-up to an experimental field study that had been conducted in the conurbation of Zurich (*11*). Along the urban periphery, we selected 12 study plots of 1-km^2^ each and 1 additional, 6-km^2^, plot. Experimental bait delivery was structured in 2 phases: April 2000–October 2001 and November 2001–December 2003. Within these 2 periods, a total of 5 different treatment schemes were used in the study plots:1 ) no bait delivery during the whole study (co/co); 2) monthly bait delivery during the first phase and trimonthly delivery during the second phase (b1/b3); 3) no bait delivery during the first phase and trimonthly delivery during the second phase (co/b3); 4) monthly bait delivery during the first phase and no delivery during the second phase (b1/co); and 5) monthly bait delivery during the first phase and the second phase (b1/b1) (Figure 1).

We used commercial fox baits (Impfstoffwerk Dessau Tornau GmbH, Rosslau, Germany) that contained 50 mg of the anthelminthic praziquantel (Droncit Bayer AG, Leverkusen, Germany). At each baiting interval, 50 baits per km^2^ were distributed manually at places that were most likely to be frequented by foxes (*11*).

Using a sandwich ELISA, we determined the effect of baiting by detecting *E. multilocularis* coproantigens in fox feces (*13*). Feces samples were collected at least once per month in all study plots during sampling period 1 before and during the initial phase of bait delivery (November 1999–June 2000), sampling period 2 (July–October 2001), and sampling period 3 (September–December 2003). Additionally, during November 2006–January 2007 (sampling period 4), feces samples were collected in the three 1-km^2^ plots that had never been baited and in the treated section of the 6-km^2^ plot that had been baited monthly throughout the entire baiting period (Figure 1).

**Figure 2:**
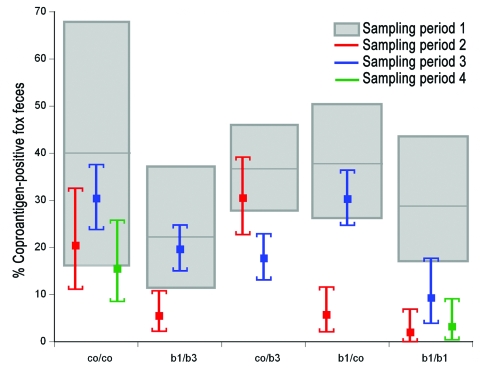
Contamination with *Echinococcus multilocularis* shown in study plots. Portion of coproantigen-positive (by ELISA) fox feces in study plots with 5 different treatment schemes (see Figure 1). Gray outlined boxes and error bars represent the 95% confidence intervals of ELISA-positive feces during the 4 sampling periods. Treatment schemes: co/co, control/control; b1/b3, monthly/trimonthly baiting; co/b3, control/trimonthly baiting; b1/co, monthly baiting/control; and b1/b1,monthly baiting/monthly baiting.

The proportion of coproantigen-positive feces detected by ELISA (hereafter referred to as *E. multilocularis* contamination) showed no significant statistical variation in the three 1-km^2^ plots that were never baited and averaged 26.5% contamination (Figure 2). In the 6 areas that were baited at monthly intervals during the first phase, *E. multilocularis* contamination decreased significantly from sampling period 1 (b1/b3 plots: 22.2%, 95% confidence interval [CI] 11.2%–37.1%; b1/co plots: 37.7%, CI 26.3%–50.2%) to sampling period 2 (b1/b3 plots: 5.4%, CI 2.4%–10.4%; b1/co plots: 5.6%, CI 2.3%–11.2%) (Figure 2). After reassigning 3 monthly baited plots to control plots (b1/co plots), a significant rise of *E.multilocularis* contamination was detected to 30.2% (CI 24.9%–36.0%). Also, the change from a monthly baiting interval to a trimonthly interval (b1/b3 plots) resulted in a significant increase to 19.5% (CI 15.2%–24.4%), whereas the trimonthly bait delivery in the co/b3 plots led to a significant decrease from 30.4% (CI 22.9%–38.8%) to 17.6% (CI 13.3%–22.5%).

Three years after the end of bait delivery, *E. multilocularis* contamination in the 3 co/co plots remained on a similar level (15.4%, CI 8.7%–24.5%). In contrast, *E. multilocularis* contamination was still low (3.1%, CI 0.6%–8.7%) in the b1/b1 plot. The 3 coproantigen-positive feces samples did not contain taeniid eggs.

## Conclusions

This 6-year experimental field study shows basic information about the susceptibility of *E. multilocularis* to small-scale control strategies targeted to high disease-endemic foci in urban areas. Monthly, local delivery of anthelmintic baits reduced *E. multilocularis* contamination to a low level. Our experimental model shows that although the trimonthly baiting intervals reduce such contamination significantly, the reduction is far less than that achieved by baiting at monthly intervals. In addition, trimonthly baiting did not help to maintain the low *E. multilocularis* contamination achieved by the previous 1.5 years of intensive monthly baiting.

Our finding contrasts with the results of 2 German studies: in rural, large-scale study areas where baits were distributed once every 3 months during 9 and 18 months, respectively, baits were effective in maintaining the prevalence of *E. multilocularis* in foxes at the same level as that in previous baiting campaigns conducted at intervals of 6 weeks (*6*,*7*). It is possible that lower fox densities in rural areas compared with urban areas or the shorter periods during which trimonthly baiting intervals were applied are responsible for this difference. More likely, the difference can be explained by border effects. Foxes with home ranges near the border of a baited area have less access to the anthelminthic baits. Furthermore, immigrating foxes can contribute to *E. multilocularis* contamination. In large baiting areas a smaller proportion of treated foxes live near the border of the treated area and a smaller proportion of foxes are immigrated foxes. Therefore, small baiting areas are more affected by border effects. Further decreasing the baiting frequency to 6-month intervals and discontinuing bait distribution caused a surge of *E. multilocularis* prevalence in foxes to the precontrol level within 36 months (*6*).

*E. multilocularis* contamination in nonbaited areas (co/co plots) was higher during period 3 than during period 4 (Figure 2). Natural fluctuations possibly contributed to generally low *E. multilocularis* prevalences during the latter period. However, it was surprising that contamination was still very low 3 years after all bait delivery was ended in an area that was baited monthly during a 3.5-year period. Before baiting started, *E. multilocularis* was highly prevalent in this plot, and prevalences in European water voles (*Arvicola terrestris*) ranged from 9% to 21% during several years (1997–2000) (*11*,*13*). We therefore assume that a long-lasting control effect was achieved by local removal of the parasite by monthly anthelmintic bait delivery over several years.

Our results confirm the high potential of *E. multilocularis* to recover to a precontrol level within a short period if the parasite was not completely removed, as has been predicted by modeling studies (*14**,**15*) and shown in a single, but large, baiting area in Germany (*6*). On the basis of a spatially explicit model, Hansen et al. recommend continued bait delivery with intervals from 4 to 6 weeks (*15*). Our results support these findings. We therefore suggest continued intense baiting even if parasite contamination has been substantially reduced. Furthermore, our results provide evidence that, once the parasite has disappeared locally, recolonization can take several years, even in a small-scale baiting area.
